# Improving the distribution and uptake of medicines: lessons from the global trachoma programme

**Published:** 2023-05-22

**Authors:** Angelia Sanders, PJ Hooper, Scott McPherson, Tim Jesudason

**Affiliations:** Chair: International Coalition for Trachoma Control, Atlanta, USA.; Vice Chair: International Coalition for Trachoma Control, Atlanta, USA.; Immediate Past Chair: International Coalition for Trachoma Control, USA.; Special Projects and Campaign Partnerships: International Coalition for Trachoma Control, London, UK.

**Access to, and uptake of, safe and effective medicine is a pillar of the World Health Organization (WHO)-endorsed SAFE strategy to eliminate trachoma as a public health problem.**

**Figure:**
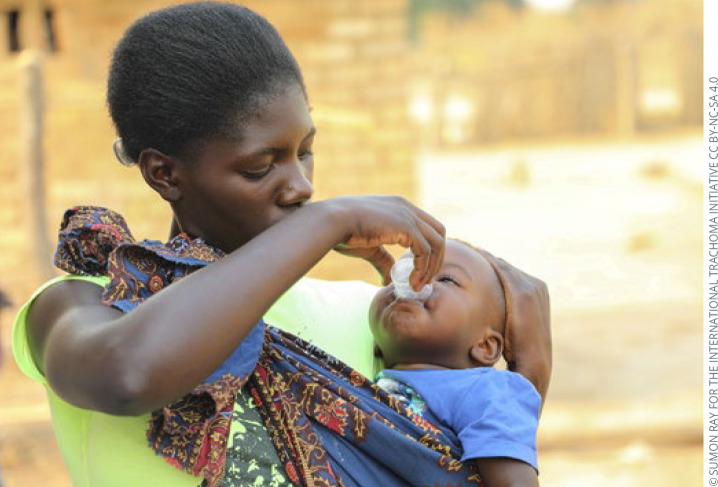
A mother administers azithromycin (Zithromax®) as a powder for oral suspension (POS) to her child. zambia

Scale-up of the SAFE strategy (surgery, antibiotics, facial cleanliness, environmental improvement), including the distribution of more than one billion doses of donated Zithromax® since 1999,^1^ has contributed to a 92% reduction in the number of people at risk of trachoma^2^ and the validation of 17 countries as having eliminated trachoma as a public health problem since 2002.^3^

As the global trachoma programme has matured, several lessons have been learned that have enabled countries to increase antibiotic coverage to effectively reduce transmission in the community and reduce the re-emergence of infection.

## Lesson 1: Supportive supervision

Supervision of community health workers and community drug distributors (CDD) is essential to ensure that optimal coverage is achieved, medicines are distributed appropriately and safely, and strategies for further performance improvement are identified.

Across the global trachoma programme, supervisors receive training according to the International Coalition for Trachoma Control preferred practice manual, titled Supportive Supervision for Mass Drug Administration with Zithromax®.

After receiving training, supervisors play an important role in trachoma teams, providing support, troubleshooting challenges experienced by CDDs, and gathering information on any cases of severe adverse events after taking the medicine.

Supervisors are also responsible for the evaluation of individual CDD performance through a supportive supervision framework, which means evaluation is conducted in order to improve the performance of the individual and that of the team. Supportive supervisors should have strong communications skills, be a team builder, and serve as a mentor.

## Lesson 2: Review programme data

The global trachoma programme regularly emphasises the power of data for improving programming. This includes carrying out systematic assessments of mass drug administration (MDA) campaigns to ensure high quality performance. All MDA campaigns are advised to collect and analyse:

1. Inventory results and accuracy
2. Coverage rates, based on districts’ distribution numbers
3. Coverage rates, based on districts’ reported leftover inventory
4. Coverage rates, based on leftover physical inventory counting.

Some programmes may also consider coverage surveys after MDA or use intra-process monitoring tools such as the Supervisory Coverage Tool to confirm reported data.

## Lesson 3: Patient safety

Global health programmes have an obligation not only to provide benefits to populations, but also to minimise harm to individuals. Zithromax® is pharmacologically safe; however, protocols must be in place to ensure prompt investigation, management, and reporting of serious adverse events, such as incidents of choking.

Ensuring that MDA programmes are implemented safely is essential to build trust with communities and sustain high MDA coverage. However, MDA safety ultimately depends on the quality of the interaction between CDDs and the person taking the medicine (or in the case of young children, the child's parent or guardian). CDDs must follow treatment guidelines and be adequately trained, prepared, and able to effectively communicate with parents and children.

The International Trachoma Initiative's Zithromax® Management Guide^4^ provides recommendations that national trachoma programmes can implement to improve patient safety. Furthermore, lessons to improve patient safety from trachoma and eye health programmes could inform other disease programmes that benefit from Zithromax®, including typhoid and yaws programmes.

## Conclusion

More than two decades of coordination and partnership across the global trachoma programme has facilitated the sharing of lessons and experiences, thereby enabling strategies to be refined to improve coverage rates and ensuring that trachoma interventions are implemented effectively, efficiently, and safely.

Notably, in 2021, more than 64 million people^2^ were reported to have received antibiotics for trachoma globally, despite ongoing challenges associated with COVID-19. Moreover, the proportion of treated districts achieving coverage of over 80% (a benchmark for acceptable coverage rates), was more than 80% globally.

Going forward, the trachoma community is continuing to share lessons to ensure that high coverage is achieved equitably. In particular, tailored strategies are being adopted so that special populations, including refugees, internally displaced persons, indigenous and nomadic populations, and people with disabilities, are not left behind within trachoma programmes. The lessons learned and documented by the trachoma community, including supportive supervision, monitoring and evaluation, and patient safety, will be essential for achieving high coverage both for trachoma and other NTDs amenable to preventive chemotherapy, to accelerate progress towards the global NTD road map targets, and to ensure that no one is left behind.
